# Pressure-support ventilation or T-piece spontaneous breathing trials for patients with chronic obstructive pulmonary disease - A randomized controlled trial

**DOI:** 10.1371/journal.pone.0202404

**Published:** 2018-08-23

**Authors:** José Augusto Santos Pellegrini, Márcio Manozzo Boniatti, Viviane Corrêa Boniatti, Crislene Zigiotto, Marina Verçoza Viana, Wagner Luiz Nedel, Leonardo da Silva Marques, Moreno Calcagnotto dos Santos, Clarissa Balbão de Almeida, Cláudia Pellizzer Dal’ Pizzol, Patrícia Klarmann Ziegelmann, Sílvia Regina Rios Vieira

**Affiliations:** 1 Department of Critical Care Medicine, Hospital de Clínicas de Porto Alegre, Universidade Federal do Rio Grande do Sul, Porto Alegre, Brazil; 2 Intensive Care Unit, Hospital Nossa Senhora da Conceição, Porto Alegre, Brazil; 3 Intensive Care Unit, Hospital Montenegro, Montenegro, Brazil; 4 Statistics Department and Post-Graduation Program in Epidemiology, Universidade Federal do Rio Grande do Sul, Porto Alegre, Brazil; Postgraduate Institute of Medical Education and Research, INDIA

## Abstract

**Background:**

Little is known about the best strategy for weaning patients with chronic obstructive pulmonary disease (COPD) from mechanical ventilation. Spontaneous breathing trials (SBT) using a T-piece or pressure-support ventilation (PSV) have a central role in this process. Our aim was to compare T-piece and PSV SBTs according to the duration of mechanical ventilation (MV) in patients with COPD.

**Methods:**

Patients with COPD who had at least 48 hours of invasive MV support were randomized to 30 minutes of T-piece or PSV at 10 cm H_2_O after being considered able to undergo a SBT. All patients were preemptively connected to non-invasive ventilation after extubation. Tracheostomized patients were excluded. The primary outcome was total invasive MV duration. Time to liberation from MV was assessed as secondary outcome.

**Results:**

Between 2012 and 2016, 190 patients were randomized to T-piece (99) or PSV (91) groups. Extubation at first SBT was achieved in 78% of patients. The mean total MV duration was 10.82 ± 9.1 days for the T-piece group and 7.31 ± 4.9 days for the PSV group (p < 0.001); however, the pre-SBT duration also differed (7.35 ± 3.9 and 5.84 ± 3.3, respectively; p = 0.002). The time to liberation was 8.36 ± 11.04 days for the T-piece group and 4.06 ± 4.94 for the PSV group (univariate mean ratio = 2.06 [1.29–3.27], p = 0.003) for the subgroup of patients with difficult or prolonged weaning. The study group was independently associated with the time to liberation in this subgroup.

**Conclusions:**

The SBT technique did not influence MV duration for patients with COPD. For the difficult/prolonged weaning subgroup, the T-piece may be associated with a longer time to liberation, although this should be clarified by further studies.

**Trial registration:**

ClinicalTrials.gov NCT01464567, at November 3, 2011.

## Introduction

Timely weaning of patients with COPD from mechanical ventilation remains a fundamental goal, although it is a difficult task. Evidence indicates that the weaning period might represent 40% of the total MV duration, [1] and weaning failures are exceedingly common in the COPD population. [2] Unfortunately, the predictors of successful weaning may not be sufficient to identify patients who are suitable for extubation, [3] and their systematic utilization could even prolong MV. [4]

The use of spontaneous breathing trials (SBTs) is the current weaning policy in more than 60% of European intensive care units (ICUs). [5] These can be performed through a variety of methods, but low levels of pressure-support ventilation (PSV) and T-piece comprise between 30% and 50% of the SBTs employed. [6] Recent guidelines suggest that PSV should be the initial choice when performing SBTs in patients who have been ventilated for more than 24 hours, [7] although this is not specifically required for the COPD population.

Our group recently published a systematic review with a meta-analysis of trials that compared PSV to a T-piece to wean patients from mechanical ventilation. [8] Of the 12 included studies, only two exclusively enrolled patients with COPD; [9, 10] in addition, we identified marked clinical heterogeneity, which prevented data pooling for this subgroup. Therefore, our primary objective was to compare the use of the T-piece and PSV for SBTs in terms of the total MV duration in patients with COPD who were being weaned from MV after being ventilated for more than 48 hours.

## Methods

### Study design

We conducted a multicenter, randomized controlled trial, which enrolled mechanically ventilated patients with COPD in southern Brazil.

### Patients

Consecutive patients with COPD were eligible after being invasively ventilated for 48 hours. We considered this interval to be necessary in order to identify patients whose indication for MV was from respiratory causes (rather than non-complicated postoperative causes). COPD diagnosis was based on clinical history, physical examination, medical records, and radiological findings. Spirometric data were not required. We excluded patients who were tracheostomized, individuals who had been previously enrolled in another trial, and patients who had previously been mechanically ventilated during the current hospitalization.

#### Patient classification

We employed International Consensus Conference Statement definitions [11], as follows: simple-weaning patients were those individuals proceeding from weaning initiation to successful extubation on the first attempt; difficult-weaning comprised those who failed initial weaning and required up to three SBT or as long as seven days from the first SBT to successful weaning; prolonged-weaning were those requiring at least three attempts or more than seven days of weaning after the first SBT.

Because of the different clinical scenarios and because time to liberation, according to our definition (see below), could be measured only for patients who failed at least one SBT, we dichotomized our analysis as patients with simple weaning versus difficult/prolonged weaning.

### Study protocol

This protocol was conducted in three medical/surgical ICUs: a 43-bed university hospital ICU (Hospital de Clínicas de Porto Alegre), a 59-bed public hospital ICU (Hospital Nossa Senhora da Conceição), and a 10-bed community hospital ICU (Hospital Montenegro). Sedation management protocols were followed at all study centers.

Clinical and epidemiological data were collected at baseline, but patient-identifying information was not collected. MV was instituted using Servo-i (Maquet SA, Ardon, France), Evita-4 (Dräeger, Lubeck, Germany) or Nellcor Puritan-Bennett 840 (Carlsbad, CA) systems.

All patients were screened daily for previously described weaning readiness criteria [11]: adequate cough and/or absence of excessive bronchial secretions, SaO2 > 90% on FiO2 < 0.5, PEEP < 8cmH2O, respiratory frequency of less than 35 breaths/min, maximal inspiratory pressure more negative than - 20cmH2O, tidal volume greater than 5 ml/kg of predicted body weight, and adequate mental status.

This study protocol followed the ethical principles of the Declaration of Helsinki [12] and of the National Health Council 466/12 resolution and was approved by the research ethics committee of Hospital Nossa Senhora da Conceição under registration number 11–234. Written informed consent was obtained from every included patient or from their next of kin.

### Randomization and allocation concealment

Sequentially-numbered, opaque, sealed envelopes were only opened once the patients fulfilled at least three of the weaning readiness criteria, and they were then considered able to perform the first SBT.

Randomization to determine the SBT modality was conducted by random sequence generation using web-based software (www.randomizer.org) in a 1:1 ratio. Block randomization with a size of 10 was performed and stratified according to the SAPS III (Simplified Acute Physiology Score, III) score.

### Interventions

The pressure support of the PSV-group patients was adjusted to 10 cm H2O while maintaining the previously adjusted positive end-expiratory pressure (PEEP) and oxygen inspired fraction–the last could be adjusted when necessary to achieve an arterial saturation of at least 92%. We adopted 10 cm H2O once it was considered a representation of our daily clinical practice and of previously published trials on this subject; [13, 14] however, some authors decided to apply lower PS levels, and [15, 16] others [10, 17] adopted even higher levels. We decided to add PEEP to PS according to physiological evidence of better tolerance to weaning in a population of patients with a high COPD prevalence. [18]

For patients in the T-piece group, the tracheal tube was disconnected from the ventilator and attached to a “T” connector, which continuously supplied humidified oxygen to achieve a saturation of at least 92% in the absence of positive pressure. Both SBTs were accomplished in 30 minutes in a semi-seated position.

We registered heart rate, pulse oximetry, and patient tolerance according to clinical evaluation at 0, 15 and 30 minutes. Respiratory rate, tidal volume, and minute ventilation were measured at 0 and 30 minutes using a handheld Wright respirometer (Ferraris Medical, Hertford, Herts, UK).

The following signs of poor spontaneous breathing tolerance were used as criteria for prematurely terminating the trial, hence defining SBT failure: respiratory rate > 35 breaths/min, oxygen saturation < 88%, heart rate > 140/min, systolic blood pressure > 200 mmHg or < 80 mmHg (or any variation exceeding 30% difference from the baseline), exaggerated use of accessory respiratory muscles, consciousness disturbance, diaphoresis or new arrhythmia. Patients who failed SBT were returned to their previous MV settings for at least 24 hours. The same SBT randomized modality was maintained for further SBTs.

All patients that tolerated the test were extubated and were preemptively connected to non-invasive ventilation (NIV, BiPAP, Vision; Philips Respironics Inc., Murrysville, PA) immediately following tracheal tube removal for a period of at least 4 hours unless contraindicated. The overall duration of NIV was determined according to clinical criteria assessed by the ICU staff. After 24 hours from extubation, we defined "Use of NIV" as a course of at least 4 hours in each following day. Each patient was considered free of NIV if attained two consecutive days without NIV use (or for less than 4 hours—e.g., during respiratory therapy sessions).

### Outcomes

The primary outcome was total MV duration, defined as the interval between the intubation day and one of the following: successful extubation (48 hours without invasive ventilatory support), death while on MV, or tracheostomy. When patients were reintubated sooner than 48 hours, we considered that the MV duration was still ongoing.

The time to liberation, defined as the interval between the day on which the first SBT was performed and one of the three aforementioned events, was evaluated as a secondary outcome. Because the time to liberation could be measured only for individuals not successfully extubated at their first SBT, this outcome was obtained only for the difficult/prolonged-weaning population. [11]

Additional exploratory outcomes were (a) the successful extubation rate at first SBT, (b) the overall reintubation rate, (c) tracheostomy incidence, (d) ICU mortality, (e) factors associated with time to liberation, and (f) factors associated with 48-hour failure rate.

### Statistical analysis

Sample size was calculated considering the mean MV duration in COPD patients at Hospital Nossa Senhora da Conceição (5.8 ± 2.44 days). Hence, the enrollment of 190 patients provided 80% power for detecting a reduction of 1 day of MV duration, considering alpha equal to 5% and using two-sided Wald test provided by the generalized linear model with Poisson error and log link function.

Normality was assessed for continuous variables using Shapiro-Wilk test. Pairwise comparisons between the two study groups were performed for physiological parameters and continuos exploratory outcomes using t-test or Wilcoxon-Mann-Whitney test according to normality criteria. Fisher’s exact test was used to compare the study groups regarding binary variables.

Primary analyses were unadjusted, intention-to-treat comparisons between the two study groups regarding the primary outcome of total MV duration. These data were positively skewed, violating the assumptions on which the usual least square models are based. Therefore, we adopted generalized linear models and specified that the data follow a gamma distribution and a log link function. This allowed a proportional impact of the T-piece strategy over PSV; therefore, the effect size measures used for the primary outcome were mean ratios. The same rationale was applied for the time to liberation secondary outcome analysis.

For the unadjusted 48-hour reintubation rate, we created Poisson linear models with robust estimation. Outputs from this analysis were then summarized as risk ratios. Likelihood ratio chi-square test was used to compare the study groups in all unadjusted analysis.

Multivariate generalized linear models were constructed to identify variables that are independently associated with weaning duration and 48-hour reintubation rate. For both outcomes, study group was maintained as the variable of interest. *A priori* defined external factors were those that have been reported in the literature to be associated with the outcomes or that are plausibly associated: age, SAPS score, hypercapnic exacerbation (here defined as having a PaCO2 more than 55mmHg on ICU admission), 24-hour pre-SBT fluid balance and pre-SBT duration. In addition, we included any variable that resulted in a *p*-value of less than 0.20 in the univariate analysis. Regression was constructed using the backward stepwise method. The Wald test was used to compare the study groups.

All the analyses were performed using IBM SPSS Statistics software, version 20.0 (IBM Corp., Armonk, NY, USA). Statistical significance was set at 0.05. Interim analysis at 6-month intervals was conducted by the investigators for security concerns.

## Results

**Fig 1:**
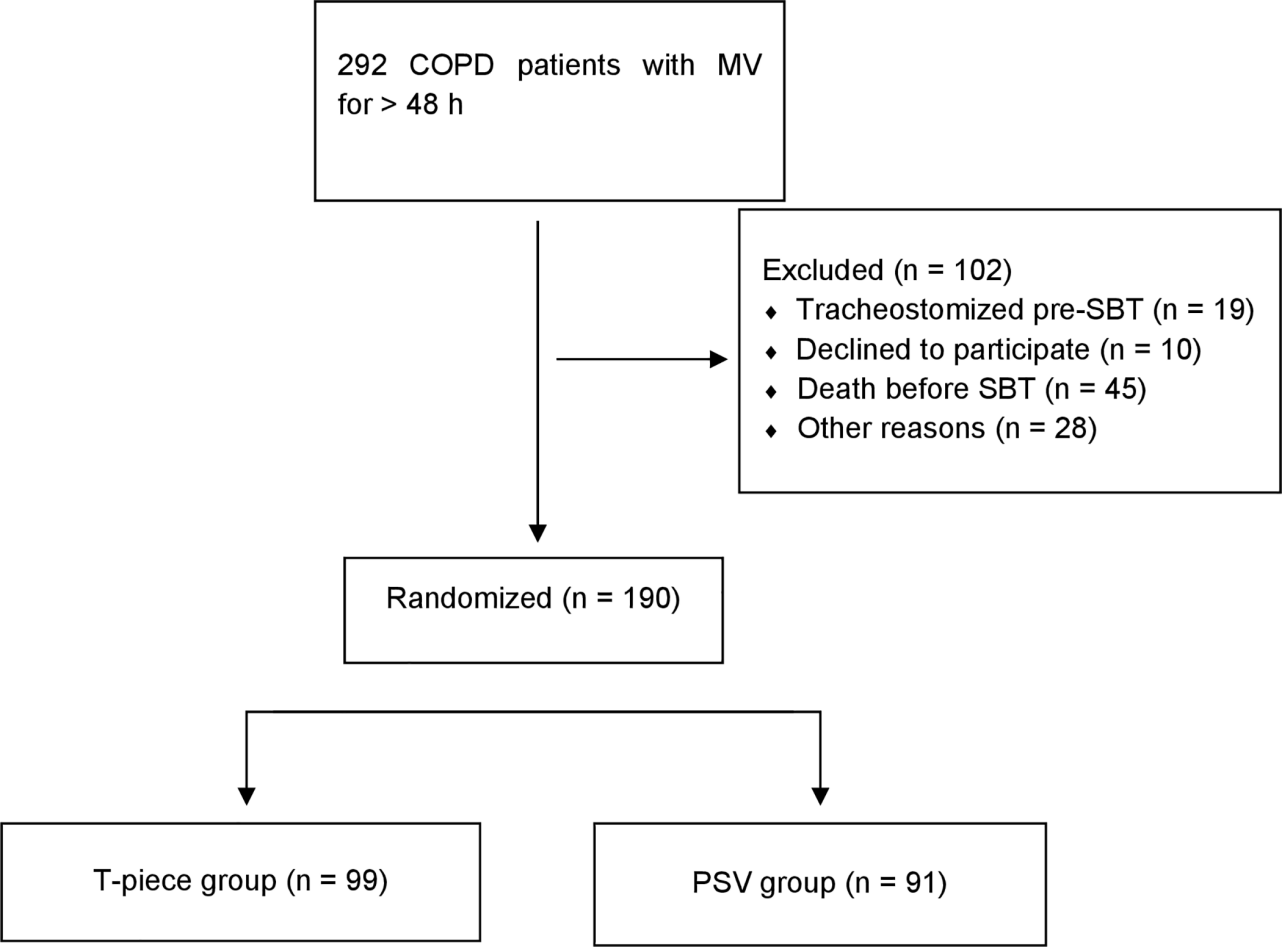
Patients enrollment and randomization.

Between 2012 and 2016, 292 patients with COPD underwent MV for more than 48 hours and were screened for eligibility. Among them, 190 patients were randomized as depicted in Fig 1; ninety-nine patients were assigned to the T-piece group and 91 patients to the PSV group.

Patient baseline characteristics are shown in Table 1. Clinical and demographic variables were similar between groups. There were no significant differences in physiological parameters either between 0 and 30 minutes of SBT performance or between study groups at 30 minutes (Table 2). Five patients (2.7%) had contraindications for NIV (somnolence = 1; facial abnormalities = 2; assistant physician disagreement = 1; NIV unavailable = 1) and hence did not undergo such therapy.

**Table 1:** Baseline characteristics of the study population.

Characteristic	T-piece (99)	PSV (91)
Age—years	67.99 ± 11.37	67.30 ± 9.83
Male sex—n (%)	52 (52.2)	50 (54.9)
SAPS III score	68.04 ± 12.36	67 ± 14.28
Hypercapnic exacerbation	69 (70.4)	55 (61.8)
24 h pre-SBT fluid balance—ml	128.52 ± 1436	375. 73 ± 1428
Setting from which the patient was admitted to the ICU—n (%)		
Emergency room	56 (57.1)	44 (49.4)
Ward	32 (32.7)	37 (41.6)
Others	10 (10.1)	8 (8.8)
Reason for mechanical ventilation—n (%)		
Exacerbation of COPD	40 (40.4)	36 (40.4)
Pneumonia	35 (35.4)	34 (38.2)
CNS depression	8 (8.1)	7 (7.9)
Post-operative	5 (5.1)	5 (5.6)
Acute decompensated heart failure	2 [19]	0 (0)
Pulmonary embolism	1 (1)	0 (0)
Other causes	8 (8,1)	7 (7,9)

Plus-minus values are means ± SD. There were no significant differences between the two groups in any of the characteristics listed. PSV, Pressure-Support Ventilation; SAPS, simplified acute physiology score; SBT, spontaneous breathing trial; ICU, intensive care unit; COPD, Chronic Obstructive Pulmonary Disease; CNS, central nervous system.

**Table 2:** Physiological Parameters at 0 and 30-minutes intervals.

Parameter	Before T-Piece (Time "0")	Before PSV (Time “0")	T-Piece Time "30"	PSV Time "30"
Respiratory rate (breaths/min)	24.17 ± 5.74	22.28 ± 7.04	24.35 ± 6.39	23.69 ± 7.45
Heart rate (beats/min)	93.83 ± 14.94	90.40 ± 16.15	95.88 ± 13.39	92.94 ± 20.19
Systolic arterial pressure (mmHg)	135.42 ± 22.20	137.44 ± 24.75	138.67 ± 25.72	139.64 ± 22.52
Arterial saturation (%)	97.48 ± 2.82	97.34 ± 2.47	96.48 ± 3.80	96.88 ± 2.74
Central venous saturation (%)	72.34 ± 9.48	71.81 ± 10.24	71.74 ± 9.89	71.20 ± 8.64
PaCO2 (mmHg)	49.46 ± 11.02	49.53 ± 13.15	-	-
PSV level (cmH2O)	12.23 ± 2.61	12.38 ± 2.46	-	-
PEEP level (cmH2O)	6 (5–7)	6 (5–7)	-	-
Minute ventilation (L/min)	8.16 ± 3.50	8.38 ± 3.56	9.65 ± 3.39	8.72 ± 3.62
RSBI (breaths/min/L)	70.21 (45.06–95.36)	69.67 (44.9–94.47)	64.80 (42.3–87.3)	63.95 (38–89.9)

Plus-minus values are means ± SD or median and (interquartile range). SBT, spontaneous breathing trial; PSV, Pressure-Support Ventilation; PaCO2: carbon dioxide partial arterial pressure; PEEP: positive end-expiratory pressure; RSBI, Rapid Shallow Breathing Index. There were no significant differences between pairwise comparisons between the two study groups at time “30" or between these and baseline (all p-values > 0·05).

Patients in the T-piece group had a longer pre-SBT interval (a variable unaffected by the intervention itself) than those in the PSV group, owing to a baseline imbalance (Table 3). The total MV duration (a variable that incorporates the pre-SBT interval) for patients in the T-piece and PSV groups was therefore also different. For patients in the difficult/prolonged weaning subgroup (n = 71, comprising 56 difficult-weaning and 15 prolonged-weaning patients), the T-piece SBT was associated with a time to liberation that was approximately doubled.

**Table 3:** Mechanical ventilation stages.

Interval	T-piece (99)	PSV (91)	Unadjusted Mean Ratio	p
Pre-SBT no. 1—days	7.35 ± 3.9	5.84 ± 3.3	1.28 (1.10–1.49)	0.002^§^
Difficult/Prolonged Weaning—n (%)	39 (39.4)	32 (35.2)	-	0.553^¶^
Time to Liberation—days ^†^	8.36 ± 11.04	4.06 ± 4.94	2.06 (1.29–3.27)	0.003^§^
Total MV duration—days	10.82 ± 9.1	7.31 ± 4.9	1.48 (1.22–1.80)	< 0.001^§^
Post-Extubation NIV–days	1.12 ± 1.66	1.18 ± 1.59	0.95 (0.77–1.23)	0.856^§^

Data are presented as means ± standard deviation or numbers (percentage). PSV, Pressure-Support Ventilation; SBT, spontaneous breathing trials; MV, mechanical ventilation; NIV, non-invasive ventilation.

§ Univariate Generalized Linear Model considering Study Group as a predictor for each outcome and PSV as a reference. p-values refer to the likelihood ratio chi-square test.

† For the difficult-weaning subgroup, n = 71.

¶ Fisher’s exact test

Additional exploratory outcomes are listed in Table 4. We did not identify significant differences between groups regarding any outcome.

**Table 4:** Exploratory outcomes.

Outcome	T-Piece (99)	PSV (91)	Risk Ratio (95% CI)	p
Extubation at first SBT—n (%)	78 (78.8)	71 (78.8)	1.01 (0.73–1.39)	0.953^§^
48-hour failure rate—n (%)	28 (29.5)	22 (24.2)	1.22 (0.70–2.13)	0.485^§^
Overall reintubation rate—n (%)	35 (36.8)	40 (44)	0.84 (0.53–1.32)	0.445^§^
Tracheostomy—n (%)	22 (22.2)	15 (16.5)	1.35 (0.70–2.60)	0.369^§^
ICU LOS—median (IQR)	13 (5–21)	11 (6–16)	-	0.465^¶^
ICU mortality—n (%)	26 (26.5)	25 (28.4)	0.93 (0.54–1.61)	0.807^§^
Hospital LOS—median (IQR)	32.5 (11.5–53.5)	32 (10–54)	-	0.971^¶^

Data are presented as numbers (percentage). PSV, Pressure-Support Ventilation; CI, confidence interval; SBT, spontaneous breathing trials; ICU, intensive care unit; LOS, length of stay; IQR, interquartile range.

^§^ Univariate Poisson regression model.

^¶^ Wilcoxon-Mann-Whitney’s test.

From 114 patients discharged alive from hospital, 28 (24.5%) were on supplemental oxygen and 9 (7.9%) were still tracheostomized. No patients were discharged on home NIV.

We constructed a generalized linear model aiming to identify variables associated with time to liberation, including only the difficult/prolonged weaning subgroup (n = 71; Table 5). In a univariate analysis, in addition to the study group, male sex was associated with longer weaning. Controlling for other important variables (Model 1), only the study group remained independently associated with time to liberation. In a post-hoc analysis, we excluded two outliers in the T-piece group, and the results remained unchanged (1.65 [1.04–2.63]; p = 0.034). We developed an additional model (Model 2) including two variables that could bias these results: tracheostomy incidence and ICU mortality. Even then, the T-piece group maintained its association with a longer weaning time.

**Table 5:** Gamma log link models for time to liberation outcome.

Variable	Weaning days (mean ± SD)	unadjusted analysis ^¶^	p-value	Adjusted—Model 1 *	p-value	Adjusted—Model 2 **	p-value
Study group							
T-piece	8.36 ± 11.04	2.06 (1.29–3.27)	0.002^†^	1.96 (1.18–3.24)	0.009^‡^	1.92 (1.21–3.03)	0.005^‡^
PSV	4.06 ± 4.94	1.00		1.00		1.00	
Sex							
Male	7.58 ± 10.53	1.71 (1.05–2.79)	0.031^†^	1.70 (0.99–2.89)	0.051^‡^	1.42 (0.85–2.35)	0.176^‡^
Female	4.42 ± 5.18	1.00		1.00		1.00	

PSV, Pressure-Support Ventilation.

* Model 1 refers to adjustment for the following variables: age, simplified acute physiology score, hypercapnic exacerbation, 24-hour pre-SBT fluid balance and pre-SBT duration.

** Model 2 refers to Model 1 plus tracheostomy and ICU death.

¶ Data are presented as mean ratios (95% CIs).

† Likelihood ratio chi-square test.

‡ Wald test.

We then aimed to identify variables that were associated with the 48-hour extubation failure rate using a Poisson regression model (Table 6). In a univariate analysis, male sex and pre-SBT duration were associated with this outcome. In a multivariate analysis, both retained an independent association; the study group, however, was not associated with this outcome in any step of this analysis.

**Table 6:** Poisson models for 48-hour failure rate outcome.

Variable	incidence—n (%)	unadjusted analysis ^¶^	p-value ^†^	adjusted analysis ^¶^ *	p-value ^‡^
Study group					
T-piece	28 (29.5)	1.32 (0.81–2.17)	0.265	1.19 (0.73–1.94)	0.478
PSV	22 (24.2)	1.00			
Sex					
Male	35 (35.0)	1.99 (1.16–3.39)	0.012	1.93 (1.14–3.26)	0.014
Female	15 (17.4)	1.00			
Pre-SBT duration	-	1.07 (1.02–1.14)	0.007	1.07 (1.01–1.14)	0.003

PSV, Pressure-Support Ventilation; SBT, spontaneous breathing trials.

* Model adjusted for the following variables: age, simplified acute physiology score score, hypercapnic exacerbation and 24-hour pre-SBT fluid balance.

¶ Data are presented as risk ratios (95% CIs).

† Likelihood ratio chi-square test.

‡ Wald test.

## Discussion

The main result of this multicenter, randomized, controlled trial was that the MV duration, although longer for patients in the T-piece group, was not determined by the SBT technique itself, considering the baseline imbalance in the pre-SBT MV duration (a pre-randomization interval) and that most patients were extubated in their first SBT.

For difficult/prolonged-weaning subgroup, T-piece SBTs were associated with a longer time to liberation. This finding, however, corresponds to a secondary outcome and a specific subgroup, although it was revealed to be consistent in multivariate analysis, even after adjusting for different models (including pre-SBT MV duration) and censoring outliers.

Sklar et al. [20] recently pointed out that PSV significantly reduces the work of breathing and pressure-time product compared to the T-piece, which could, in turn, more closely represent the post-extubation scenario. However, NIV dissemination as an adjunctive for extubation makes clinical interpretation of these data difficult. In fact, the T-piece workload might be hard to overcome for some patients who could achieve successful extubation in other ways. This is even more important as the severity of a patient’s illness increases. For patients with COPD and difficult/prolonged weaning, the T-piece is the most demanding trial applied for this frail population, [21] and this combination may cause unfavorable outcomes.

Some authors previously reported that PSV could be superior to the T-piece for a heterogeneous critically ill population. Brochard et al. [22] compared three gradual strategies for difficult-weaning patients, including T-piece and PSV, and reported a lower failure rate and a significantly shorter time to liberation using PSV. However, Esteban et al. [23] found that once-daily T-piece SBTs resulted in faster weaning compared to PSV. These trials included SBTs as part of gradual weaning protocols; thus, it is difficult to incorporate their results in our current practice, where SBTs have gained a central role, even becoming a cornerstone of the extubation decision-making process.

More recently, Matic et al. [10] randomized exclusively patients with COPD who were ventilated for more than 24 hours using PSV (n = 70) or a T-piece (n = 66). For patients who had at least one trial failure, PSV proved to be superior to the T-piece in terms of time to liberation, MV duration, and length of ICU stay. Overall, these results are consistent with ours, notwithstanding the fact that their study was unicentric and that the authors did not standardize post-extubation NIV utilization, did not report its incidence, and did not adjust their results for potential confounders.

Preemptive use of post-extubation NIV for all randomized patients is a particular aspect of our methodology. Although not uniformly adopted by previous studies assessing weaning of patients with COPD [10, 24], this strategy is becoming more frequent [25, 26] and is now strongly recommended by international guidelines to prevent extubation failure in patients with COPD, hypercapnia or other serious comorbidities [7]. Therefore, we believe that this reflects current evidence-based medical practice and did not result in biased influence over one of the study groups.

The pre-SBT interval was independently associated with the 48-hour weaning failure rate. This could be attributable to several explanations: first, this could be simply a marker of severity, reflecting ventilatory demand, infection acquisition, and refractory bronchospasm, aspects correlated with worse overall prognosis; second, MV duration increases consequent to a pre-SBT interval increase, resulting in muscle atrophy, the need for sedation, and several unintended outcomes, contributing to higher extubation failure rates; third, the physicians’ excessive caution in proceeding with weaning these individuals might be counterproductive and inconsistent with a tendency for systematic and protocol-based weaning.

Male sex was associated with the 48-hour failure rate. Previous studies noted sex-specific behaviors according to the susceptibility for the development, severity of, and response to management, [27] with a greater prevalence and mortality among men. Tobacco use patterns and genetic [28] and hormonal [29] factors may all play a role in these discrepancies. To the best of our knowledge, our results concerning a sex-specific profile for critically ill patients with COPD are still unique.

We report high reintubation and mortality rates. Patients included in our study are severely ill, as indicated by advanced mean age, predominance of hypercapnic exacerbation, and high SAPS III scores. Nevertheless, other trials reported similar reintubation rates [30]. The mean SAPS III of our population indicates a predicted hospital mortality rate exceeding 75%. Our ICU mortality rate of 28% is higher than some [10, 31], but comparable to other studies [32, 33]. These last studies reported severity scores indicating lower predicted mortality rates (APACHE II scores of 12 to 25, resulting in a predicted mortality varying from 20 to 40%).

### Strengths and limitations

Our study has several limitations. First, a causal imbalance in the pre-SBT duration precluded our ability to draw definitive conclusions concerning total MV duration. Therefore, we decided to include a pre-SBT interval in every multivariate run, drawing conclusions from other relevant outcomes. Second, the outcome definitions are not uniform between the weaning and SBT studies. More frequently than hard outcomes such as mortality or ICU and/or hospital stay, these trials assessed endpoints such as weaning success, reintubation rate, and the duration of ventilatory support according to the stage of weaning. [8] We, as others, [10, 22, 23] decided to focus on the expeditiousness of the weaning process. The duration of MV support is a widely adopted parameter of weaning efficiency and overall quality of care in terms of preventing ventilator-associated events and cost rationalization. [34] Third, the time to liberation was essentially a secondary outcome, and the difficult/prolonged-weaning subgroup comprised only a small fraction of the study population; thus, our results concerning the SBT strategy are not generalizable for the entire ventilated COPD population. In addition, spirometric data were not required for the diagnosis of COPD, raising the possibility that some patients could have other clinical conditions mimicking it. However, this approximates what is encountered in clinical practice, where many patients with a diagnosis of COPD are managed based on clinical presentation. Finally, the chosen PSV level is higher than in other studies in general population [15, 16], what could be related to some degree of overassistance. For patients with COPD, however, the ideal PSV level is still unknown. Nevertheless, one shall expect that an overassisted SBT would result in inappropriately premature extubations–manifested by higher reintubation rates–what we have not identified in this population.

The study also had numerous strengths: this was a multicenter study, reflecting different organizational policies and patient profiles and should be considered representative of university, community, and public hospital populations. NIV standardization ensured that our results were not influenced by this adjunctive intervention because it was adopted in every included patient unless contraindicated. Moreover, we believe that NIV institution did not bias extubation decision, which was made adopting strictly predefined criteria for passing an SBT. This protocol was conducted in a rigorous way and was focused on reflecting actual weaning practices in the recruiting ICUs. All centers already had incorporated daily sedation and weaning readiness-screening protocols, facilitating the definition of strict criteria for SBT performance as well as for premature trial termination. Lastly, multivariate analyses were rigorously conducted using the most appropriate tools depending on data behavior.

## Conclusions

In this multicentric, randomized controlled trial, the SBT technique did not influence MV duration for patients with COPD. The T-piece was, however, independently associated with a longer time to liberation in the difficult/prolonged-weaning COPD subgroup, while not influencing the overall or 48-hour reintubation rates. PSV SBT may be considered a reasonable strategy for these severely ill patients, although further studies are warranted to corroborate this hypothesis.

## Supporting information

S1 File CONSORT checklist. (DOC)

S2 File Study protocol–original language. (DOCX)

S3 File Study protocol–English version. (DOCX)

## Abbreviations

BiPAP: BiLevel Positive Airway Pressure
COPD: Chronic Obstructive Pulmonary Disease
ICU: Intensive Care Unit
MV: Mechanical Ventilation
NIV: Non-Invasive Ventilation
PEEP: Positive End-Expiratory Pressure
PSV: Pressure-Support Ventilation
SAPS: Simplified Acute Physiology Score
SBT: Spontaneous Breathing Trial
