# Supplementary material for: Pressure-support ventilation or T-piece spontaneous breathing trials for patients with chronic obstructive pulmonary disease - A randomized controlled trial
Source: PLoS One. 2018 Aug 23;13(8):e0202404. doi: 10.1371/journal.pone.0202404 (PMC6107186; doi:10.1371/journal.pone.0202404)
Supplement: S2 File — (DOCX) [file pone.0202404.s002.docx]

GRUPO HOSPITALAR CONCEIÇÃO

HOSPITAL NOSSA SENHORA DA CONCEIÇÃO

UNIDADE DE TERAPIA INTENSIVA

**COMPARAÇÃO ENTRE TESTES DE VENTILAÇÃO ESPONTÂNEA ATRAVÉS DE PRESSÃO DE SUPORTE OU TUBO “T” NA DESCONTINUAÇÃO DA VENTILAÇÃO MECÂNICA EM PACIENTES PORTADORES DE DOENÇA PULMONAR OBSTRUTIVA CRÔNICA**

**Pesquisadores Reponsáveis:**

**José Augusto Santos Pellegrini**

**Márcio Manozzo Boniatti**

**Moreno Calcagnotto dos Santos**

**Sílvia Regina Rios Vieira**

**Viviane Martins Corrêa**

Finalidade: Pesquisa Institucional

Locais de Realização:

Unidade de Terapia Intensiva Adulto do HNSC

Unidade de Terapia Intensiva Adulto do Hospital de Clínicas de Porto Alegre

Unidade de Terapia Intensiva do Hospital Montenegro

**Porto Alegre, Julho de 2014**

**Sumário:**

1. Resumo ______________________________________________________________________ Pág 3

2. Fundamentação Teórica _________________________________________________________ Pág 4

3. Objetivos _____________________________________________________________________ Pág 6

3.1 Objetivo Primário ______________________________________________________________ Pág 6

3.2 Objetivo Secundário ____________________________________________________________ Pág 6

4. Justificativa ____________________________________________________________________Pág 7

5. Método _______________________________________________________________________ Pág 8

5.1 Local do Estudo ______________________________________________________________ Pág 8

5.2 Pacientes ___________________________________________________________________ Pág 8

5.2.1 Critérios de Inclusão _________________________________________________________ Pág 8

5.2.2 Critérios de Exclusão _________________________________________________________ Pág 8

5.2.3 Delineamento _______________________________________________________________ Pág 8

5.2.4 Coleta dos Dados e Randomização ______________________________________________ Pág 9

5.2.5 Análise Estatística ___________________________________________________________ Pág 10

5.2.6 Cálculo do Tamanho Amostral _________________________________________________ Pág 10

6. Aspectos Éticos _______________________________________________________________ Pág 11

7. Divulgação __________________________________________________________________ Pág 12

8. Orçamento __________________________________________________________________ Pág 13

9. Cronograma _________________________________________________________________ Pág 14

10. Referências ________________________________________________________________ Pág 15

11. Anexos ____________________________________________________________________ Pág 16

11.1 Termo de Compromisso para Entrega do Relatório Semestral ou Final __________________ Pág 16

11.2 Termo de Compromisso para Utilização de Dados ou Prontuários ______________________ Pág 17

11.3 Termo de Ciência do Coordenador de Área onde será realizada a pesquisa ______________ Pág 18

11.4 Lista de Checagem de Documentos _____________________________________________ Pág 19

12. Apêndices __________________________________________________________________ Pág 20

12.1 Termo de Consentimento Livre e Esclarecido ______________________________________ Pág 20

12.2 Ficha de Coleta de Dados _____________________________________________________ Pág 22

1. **Resumo:**

*Fundamentação*: A descontinuação da ventilação mecânica (VM) é parte essencial no cuidado de pacientes portadores de Doença Pulmonar Obstrutiva Crônica (DPOC) quando criticamente enfermos. A melhor estratégia a ser empregada não está estabelecida. *Objetivo*: Comparar o Teste de Ventilação Espontânea (TVE) em Pressão de Suporte com o TVE por meio de Tubo “T” na descontinuação da VM em pacientes portadores de DPOC. *Delineamento*: Ensaio Clínico. *Métodos*: Serão incluídos no estudo pacientes portadores de DPOC, transferidos à Unidade de Terapia Intensiva do Hospital Nossa Senhora da Conceição, que sejam submetidos a VM por, no mínimo, 48 horas. Quando considerados pela equipe assistente aptos para TVE, serão randomizados para uma das estratégias: TVE em Pressão de Suporte ou TVE em Tubo “T”. O Desfecho Primário do estudo será avaliar a redução do tempo de VM. Outros desfechos de interesse serão mortalidade, sucesso de extubação, tempo da descontinuação da VM, tempo de internação na UTI e incidência de traqueostomia. Registro *ClinicalTrials.gov* NCT01464567.

**2. Fundamentação Teórica:**

A Doença Pulmonar Obstrutiva Crônica – DPOC – é uma entidade nosológica cuja prevalência mantém-se elevada no Brasil e no mundo ^[[1]](#endnote-1)^ ^[[2]](#endnote-2)^, estando atualmente entre as principais causas de mortalidade ^[[3]](#endnote-3)^. Entre os pacientes portadores da doença em seu estágio avançado, Insuficiência Respiratória Aguda é o principal motivo de transferência à Unidade de Terapia Intensiva (UTI), requerendo, em grande parte dos casos, instituição de Ventilação Mecânica (VM) invasiva.

A VM é fundamental no suporte de vida quando em vigência de falência respiratória; existem, no entanto, parefeitos inerentes, em especial no grupo de indivíduos portadores de DPOC. Por tratar-se de uma condição associada a obstrução não-completamente reversível das vias aéreas, a VM pode resultar em potencialização do alçaponamento aéreo, tendo como possíveis consequências barotrauma, comprometimento hemodinâmico e aumento da sobrecarga imposta à musculatura respiratória ^[[4]](#endnote-4)^. Desta maneira, tão logo esteja resolvida a causa específica que desencadeou a instituição de VM nos pacientes com DPOC, deve-se dar início ao processo de descontinuação da VM.

O processo de descontinuação da VM consiste na retirada gradual do suporte ventilatório até que o paciente esteja apto a retomar a ventilação espontânea. A avaliação das reais condições de que dispõe o paciente para prosseguir com a retirada do suporte ventilatório passa pelos Testes de Ventilação Espontânea (TVE). Não existem indicadores específicos que determinem em que momento precisamente devem ter início os TVE. Caso iniciem-se muito precocemente, podem resultar em fadiga respiratória; caso tardem a ter início, podem acarretar atrofia da musculatura respiratória, aumento da taxa de Pneumonia Associada a Ventilação Mecânica e aumento do tempo de internação na UTI. Estima-se que o processo de descontinuação da VM pode ser responsável por até 40% do tempo total de VM; nos indivíduos portadores de DPOC, esta parcela pode ser de até 59%^[[5]](#endnote-5)^.

Os TVE podem ser realizados de diferentes formas. Pode-se utilizar o dispositivo conhecido como Tubo “T”, que permite ventilação espontânea, desconectada do ventilador, ao mesmo tempo que fornece oxigenoterapia suplementar. Tal método pode ocasionar aumento do trabalho respiratório por meio de incremento da resistência de via aérea imposta pelo diâmetro interno do tubo orotraqueal. Como maneira de contrabalaçar este efeito, pode-se lançar mão de implementação de Pressão de Suporte (PS) em níveis entre 5 e 10cmH2O, suficientes para contrapor tal aumento do trabalho respiratório.

Esteban e colaboradores ^[[6]](#endnote-6)^, em 1995, compararam 4 métodos de TVE em 130 pacientes (32% portadores de DPOC) alocados de forma randômica para TVE em Ventilação Mandatória Intermitente, TVE em PS, TVE diário em Tubo “T” ou múltiplos TVEs ao dia em Tubo “T”. Os testes em Tubo “T”, diários ou repetidas vezes ao longo do dia, permitiram extubação de forma mais rápida do que em PS ou em Ventilação Mandatória Intermitente, resultando em menor tempo de VM. Brochard e colaboradores ^[[7]](#endnote-7)^, conduziram outro ensaio clínico semelhante, em que 109 pacientes foram alocados para 3 métodos de TVE, incluindo Tubo “T” e PS. Estes autores encontraram um menor número de falhas com TVE em PS do que em Tubo “T”. Ambos os estudos concordaram no achado de que a estratégia baseada em Ventilação Mandatória Intermitente resulta em desfechos menos favoráveis. Outros ensaios clínicos mais recentes ^[[8]](#endnote-8)^^[[9]](#endnote-9)^^[[10]](#endnote-10)^ sugerem que as estratégias de TVE em Tubo “T” ou em PS podem ter desfechos semelhantes.

No contexto específico de pacientes portadores de DPOC, dois estudos recentes compararam estratégias de TVE. Matic e colaboradores, em recente ensaio clínico que incluiu 63 pacientes já com falha a um primeiro TVE, encontraram menor tempo de internação em UTI com o uso de PS em comparação com TVE em Tubo “T”. Esteban e colaboradores verificaram uma diferença sem significância estatística favorecendo PS em 50 pacientes no seu primeiro TVE.

Sendo assim, não está definida qual a melhor estratégia para otimizar o processo de descontinuação da VM em pacientes portadores de DPOC, o que torna necessária a realização de um ensaio clínico com suficiente tamanho amostral e rigor metodológico para responder de maneira adequada a esta questão.

**3. Objetivos:**

**3.1 Primário:**

Comparar o teste de ventilação espontânea em Pressão de Suporte com o teste de ventilação espontânea em Tubo “T” quanto ao tempo total de ventilação mecânica, em pacientes portadores de DPOC, submetidos a ventilação mecânica por mais de 48 horas.

**3.2 Secundários:**

Comparar, entre os testes referidos, a incidência dos seguintes desfechos de interesse:

- Tempo de Internação na UTI;

- Tempo do processo de descontinuação da VM;

- Taxa de falhas de extubação ⁄ Retorno à VM;

- Mortalidade;

- Incidência de Traqueostomia;

**4. Justificativa:**

A DPOC é uma das comorbidades mais frequentemente encontradas nos pacientes portadores de Insuficiência Respiratória Aguda hospitalizados na UTI, estando associada com risco para ventilação prolongada, aumento do tempo de internação na UTI, e falhas do processo de descontinuação da VM.

Uma vez que o risco de falha após uma extubação sem TVE pode chegar a 40%, faz-se necessário uma avaliação criteriosa de quais pacientes estão aptos à ventilação espontânea, o que pode ser realizado de diferentes formas, sendo as mais comumente utilizadas a ventilação em PS com baixos níveis ou a ventilação espontânea em Tubo “T”.

Se, de um lado, o TVE em Tubo “T” parece identificar com maior especificidade aqueles pacientes realmente aptos à ventilação espontânea e extubação, ela também pode precipitar fadiga em pacientes com obstrução ao fluxo aéreo, por aumento da resistência de via aérea, o que pode retardar a extubação destes indivíduos. De outra maneira, o TVE com o auxílio da PS pode atenuar a resistência de via aérea e o trabalho respiratório, mas pode não reproduzir fielmente as condições de uma ventilação espontânea de fato.

Pelos dados confllitantes e inconclusivos à disposição na literatura, e pela relevância da questão clínica, justifica-se a realização deste estudo.

**5. Método:**

**5.1 Locais do Estudo:**

Unidade de Terapia Intensiva do Hospital Nossa Senhora da Conceição.

Unidade de Terapia Intensiva do Hospital de Clínicas de Porto Alegre

Unidade de Terapia Intensiva do Hospital Montenegro.

**5.2 Pacientes:**

**5.2.1 Critérios de Inclusão:**

Serão incluídos no estudo, consecutivamente, pacientes portadores de DPOC, maiores de 18 anos, admitidos nas UTIs participantes do estudo, que forem submetidos a ventilação mecânica invasiva por, no mínimo 48 horas e, no máximo, 14 dias.

O diagnóstico de DPOC será realizado por meio de história clínica disponível em prontuário médico, exames complementares realizados e informações da equipe assistente.

**5.2.2 Critérios de Exclusão:**

- Presença de Traqueostomia previamente ao início do processo de descontinuação da ventilação mecânica;

- Menores de 18 anos;

- Outra indicação de ventilação mecânica que não Insuficiência Respiratória Aguda (depressão do nível de consciência, pós-operatório);

- Negativa em fornecer consentimento esclarecido;

- Indivíduos alocados em outro ensaio clínico.

**5.2.3 Delineamento:**

Ensaio Clínico.

Este estudo encontra-se catalogado na base de dados ClinicalTrials.gov sob o registro NCT01464567.

**5.2.4 Coleta dos Dados e Randomização:**

A inclusão do paciente no estudo se dará no momento em que se completarem 48 horas da instituição da ventilação mecânica, tão logo seja fornecido o consentimento esclarecido. Os dados serão então obtidos mediante anotação em fichas de coleta preservando a identidade do indivíduo.

Assim que tome início o processo de descontinuação da ventilação mecânica, a partir de agora definido como o momento em que se dará o primeiro Teste de Ventilação Espontânea, os pacientes serão randomizados para TVE em PS ou TVE em Tubo”T”. A randomização será feita através de envelopes lacrados e estratificação prévia utilizando o escore de gravidade SAPS 3 ^[[11]](#endnote-11)^.

Os pacientes alocados para TVE em PS terão o nível de Pressão reduzido para 10cmH2O, como forma de atenuar a resistência imposta pelo circuito do ventilador e tudo traqueal. Os pacientes alocados para TVE em “Tubo T” terão o tubo traqueal desconectado do ventilador e adaptado a um conector em forma de “T”, que permite a conexão de uma fonte de Oxigênio Suplementar, bem como possui outra extremidade que permanece em contato com o ar ambiente.

Serão monitorizadas de forma contínua e registradas antes e depois dos testes as Frequências Cardíaca e Respiratória, Pressão Arterial e Oximetria de Pulso.

Todos os TVEs serão realizados e acompanhados por Viviane Martins Corrêa, fisioterapeuta da UTI do HNSC e pesquisadora deste estudo, ou por José Augusto Santos Pellegrini, médico intensivista do Hospital de Clínicas de Porto Alegre e do Hospital Montenegro.

Os TVE em ambos os grupos terão duração de 30 minutos ^[[12]](#endnote-12)^.

Serão extubados aqueles pacientes que forem considerados aptos ao final do TVE segundo avaliação clínica, a critério da equipe médica assistente. Aqueles que não forem considerados aptos à extubação retornarão para VM a critério da equipe assistente, não realizando novo TVE antes das próximas 24 horas.

Em caso de falha, assim que novamente considerado apto, o indivíduo será submetido a um novo TVE mantendo-se a estratégia para a qual foi alocado, até um máximo de 3 falhas, momento em que passa a ser permitido o cruzamento entre os grupos.

Assim que extubados, todos os pacientes serão colocados em Ventilação Mecânica Não-Invasiva, refletindo a prática corrente da unidade assistencial, como forma de prevenir a fadiga ventilatória pós-extubação, seguindo as recentes evidências disponíveis.

Os pacientes serão acompanhados ao longo da internação hospitalar para verificação e registro dos referidos desfechos de interesse.

**5.2.5 Análise Estatística:**

Os dados serão armazenados e processados no Software SPSS Statistics 18.0. As variáveis contínuas serão apresentadas na forma de média e desvio padrão ou de mediana; para sua comparação, será utilizado o Teste t de Student para amostras independentes, quando possuírem distribuição normal.

Para as variáveis contínuas não-paramétricas, será utilizado o Teste t de Wilcoxon. Com relação às variáveis categóricas, será utilizado o Teste Qui-Quadrado. Quando não se satisfizerem seus pré-requisitos, será utilizado o Teste Exato de Fisher.

Será utilizado como nível de significância estatística uma diferença encontrada menor do que 0,05, bicaudal.

Serão realizadas análises interinas programadas semestralmente, como forma de preservar a segurança dos indivíduos alocados no presente estudo.

**5.2.6 Cálculo do Tamanho Amostral:**

Levando-se em consideração que o tempo médio de ventilação mecânica em indivíduos portadores de DPOC na Unidade de Terapia Intensiva do Hospital Nossa Senhora da Conceição, que permanecem em VM por mais de 48 horas é de 5,8 dias, com Desvio-Padrão de 2,44 (dados não-publicados), seria necessária a inclusão de 95 pacientes em cada grupo do estudo para que se verifique uma redução de 1 dia de ventilação mecânica, com um poder do estudo de 80%.

**6. Aspectos Éticos:**

O projeto terá início apenas após a apreciação e aprovação do Comitê de Ética em Pesquisa do Grupo Hospitalar Conceição.

Os pacientes somente serão incluídos no estudo após a assinatura do Termo de Consentimento Livre e Esclarecido, que será apresentado em duas vias, sendo que uma delas será fornecida ao paciente ou seu responsável.

O autor deste projeto garante confidencialidade quanto aos dados pessoais obtidos, assegurando como seu fim único e exclusivo a pesquisa clínica em questão. Todas as informações serão analisadas de modo agregado, preservando a confidencialidade e a identidade dos participantes. Os dados serão utilizados apenas para esta pesquisa, serão armazenados por um período de cinco anos e, após, destruídos.

Todos os aspectos éticos envolvidos encontram-se de acordo com a resolução CNS 196/96 (DO. N0 201, 16 de outubro de 1996) e suas complementares. Os investigadores comprometem-se em zelar pela *beneficência*, comprometendo-se com o máximo de benefícios e o mínimo de danos e riscos, e pela *não maleficência,* garantindo que danos previsíveis serão evitados. Asseguramos que os procedimentos propostos respeitarão a confidencialidade e a privacidade, a proteção da imagem e a não estigmatização, garantindo a não utilização das informações em prejuízo dos pacientes do estudo, inclusive em termos de auto-estima, de prestígio e/ou econômico–financeiro.

Cabe esclarecer que as medidas a serem adotadas no ambiente do estudo já são implementadas na prática clínica do cuidado diário destes pacientes, quando do processo de descontinuação da ventilação mecânica, ainda que em ambiente não-controlado.

**7. Divulgação:**

Os resultados do estudo serão submetidos à apreciação de periódicos internacionais especializados em Medicina Intensiva e Ventilação Mecânica, com vistas a publicação de relevante impacto científico.

Será entregue um exemplar da pesquisa concluída ao Centro de Documentação do Grupo Hospitalar Conceição para consulta.

**8. Orçamento:**

Não há previsão de gastos com exames laboratoriais e/ou medicações. Não serão solicitados exames laboratoriais nem será feita prescrição de medicação fora da prática clínica diária. A única intervenção será a realização dos TVEs conforme explicitado. Os gastos previstos serão com material de escritório

Não será solicitado fomento adicional às entidades de incentivo à pesquisa.

Tabela 1:

| **Material** | **Valor unitário (R$)** | **Estimativa total** | **Valor total (R$)** |
| --- | --- | --- | --- |
| Folhas A4 | 0,05 | 2000 | 100,00 |
| Cartucho impressora | 40,00 | 1 | 40,00 |
| Canetas | 2,50 | 4 | 10,00 |
| Total |  |  | 150,00 |

**9. Cronograma:**

|  | **2012** | **2013** | **2014** | **2015** |
| --- | --- | --- | --- | --- |
| **Atividade** / **Mês** | Fev a Dez | Jan a Dez | Jan a Dez | Jan a Mar |
| Inclusão de pacientes | **X** | **X** | **X** |  |
| Análise dos dados |  |  | **X** | **X** |
| Elaboração e envio de artigos científicos |  |  |  | **X** |

**10. Referências:**

1. # Benseñor IM, Fernandes TG, Lotufo PA. Chronic obstructive pulmonary disease in Brazil: mortality and hospitalization trends and rates, 1996-2008. [Int J Tuberc Lung Dis.](http://www.ncbi.nlm.nih.gov/pubmed/21333110) 2011 Mar;15(3):399-404.

   [↑](#endnote-ref-1)
2. Dolan S, Varkey B. Prognostic factors in chronic obstructive pulmonary disease. Curr Opin Pulm Med. 2005;11:149-52. [↑](#endnote-ref-2)
3. World Health Organization. World Health Report. Geneva: World Health Organization; 2000 [↑](#endnote-ref-3)
4. Tzoufi M, Mentzelopoulos SD, Roussos C, Armaganidis A. The effects of nebulized salbutamol, external positive end expiratory pressure, and their combination on respiratory mechanics, hemodynamics, and gas exchange in mechanically ventilated chronic obstructive pulmonary disease patients.

   Anesth Analg. 2005;101:843-50 [↑](#endnote-ref-4)
5. Kuhlen R, Max M. Weaning from mechanical ventilation [in German]. Anaesthesist. 1998;47:693-703. [↑](#endnote-ref-5)
6. Esteban A, Frutos F, Tobin MJ, et al. A comparison of four methods of weaning patients from mechanical ventilation. Spanish Lung Failure Collaborative Group. N Engl J Med 1995; 332:345–350 [↑](#endnote-ref-6)
7. Brochard L, Rauss A, Benito S, et al. Comparison of three methods of gradual withdrawal from ventilatory support during weaning from mechanical ventilation. Am J Respir Crit Care Med 1994; 150:896–903 [↑](#endnote-ref-7)
8. Esteban A, Alia I, Gordo F, et al. Extubation outcome after spontaneous breathing trials with T-tube or pressure support ventilation. The Spanish Lung Failure Collaborative Group. Am J Respir Crit Care Med 1997; 156:459–465 [↑](#endnote-ref-8)
9. Farias JA, Retta A, Alia I, et al. A comparison of two methods to perform a breathing trial before extubation in pediatric intensive care patients. Intensive Care Med 2001; 27:1649–1654 [↑](#endnote-ref-9)
10. Jones DP, Byrne P, Morgan C, et al. Positive end-expiratory pressure vs. T-piece. Extubation after mechanical ventilation. Chest 1991; 100:1655–1659 [↑](#endnote-ref-10)
11. Moreno RP, Metnitz PGH, Almeida E, et al: SAPS 3 - From evaluation of the patient to the evaluation of intensive care unit. Part 2: Development of a prognostic model for hospital mortality at ICU admission. *Intensive Care Med* 2005; 31:1345-1355 [↑](#endnote-ref-11)
12. Esteban A, Alía I, Tobin MJ. Effect of spontaneous breathing trial duration on outcome of attempts to discontinue mechanical ventilation. Spanish Lung Failure Collaborative Group. Am J Respir Crit Care Med. 1999;159(2):512 [↑](#endnote-ref-12)
