# Supplementary material for: Pressure-support ventilation or T-piece spontaneous breathing trials for patients with chronic obstructive pulmonary disease - A randomized controlled trial
Source: PLoS One. 2018 Aug 23;13(8):e0202404. doi: 10.1371/journal.pone.0202404 (PMC6107186; doi:10.1371/journal.pone.0202404)
Supplement: S3 File — (DOCX) [file pone.0202404.s003.docx]

**A COMPARISON BETWEEN SPONTANEOUS BREATHING TRIALS THROUGH PRESSURE-SUPPORT OR T-PIECE FOR WEANING PATIENTS WITH CHRONIC OBSTRUCTIVE PULMONARY DISEASE FROM MECHANICAL VENTILATION**

Institutional affiliations:

Hospital Nossa Senhora da Conceição

Hospital de Clínicas de Porto Alegre

Hospital Montenegro

**Porto Alegre, 2014**

1. **Abstract:**

*Background*: Liberation from mechanical ventilation is an essential component in caring critically ill patients with Chronic Obstructive Pulmonary Disease (COPD). *Objective*: To compare spontaneous breathing trials (SBT) performed with pressure-support (PS) with T-Piece for weaning patients with COPD from mechanical ventilation. *Design*: randomized controlled trial. *Methods*: Patients with clinical diagnosis of COPD, admitted to intesive care unit (ICU) and mechanically ventilated for more than 48 hours will be included. When considered able to test spontaneous breathing, according to institutional protocols, patients will be randomized for SBT through PS or T-piece. Primary outcome will be duration of mechanical ventilation. Other assessed outcomes will be ICU mortality, extubation success, weaning duration, ICU length of stay and tracheostomy incidence. *ClinicalTrials.gov* Register NCT01464567.

**2. Background:**

Chronic Obstructive Pulmonary Disease (COPD) is a prevalent nosologic entity, being among the most frequent ICU mortality causes worldwide. Among patients with COPD, acute respiratory failure is the main reason for ICU admission, warranting, commonly, invasive MV.

MV is an essential component of life support when respiratory failure presents. There are, however, inherent adverse consequences specific to the patients with COPD. Once these patients have dynamic flow obstruction, MV can result in air trapping, barotrauma, hemodynamic compromise and respiratory muscle fatigue. Likewise, as soon as the primary condition that resulted in respiratory failure is controlled, one must consider starting MV liberation.

Liberation from MV consists on gradual ventilatory support withdrawal until the patient is considered able to resume unassisted ventilation. Spontaneous Breathing Trials (SBT) are a fundamental step to assess actual conditions that patients have to proceed ventilatoy withdrawal. Specific parameteres to define precisely when to start SBTs are unavailable. If made too early, SBTs may result in respiratory fatigue; if too late, may coexist with muscle deconditioning, ventilator-associated pneumonia and increased ICU length of stay. It is estimated that MV liberation process can be responsible for over 40% of the total duration of MV; for patients with COPD, this interval could be even higher.

SBTs can be done through different techniques. One can use a T-piece connector attached to an enriched oxygen supply, while allowing spontaneous ventilation, disconnected from the ventilator. This method is associated with increased work of breathing through the increased airway resistance imposed by the internal diameter of the endotracheal tube, unassisted of positive pressure.

For counterbalancing this mechanism, one can use positive pressure to overcome this effect. SBTs using Pressure-Support (PS) are performed with positive inspiratoy pressures between 5 and 10cmH2O, theoretically sufficient to copensate the increase in the work of breathing.

Estaben et al compared 4 methods of performing SBTs in 130 patients (32% of whom with COPD), randomly allocated to Intermittent Mandatory Ventilation, PS, once-a-day T-piece or multiple T-piece during the day. T-piece SBTs allowed faster extubation and less MV duration. Brochard et al drawn a similar study, in which 109 patients were allocated to 3 different SBTs, including T-piece and PS. These authors reported lower extubation failure rates with PS than with T-piece. Both studies indicated that Intermittent Mandatory Ventilation results in less favorable outcomes. Other recent trials suggest that PS and T-piece have similar results.

Regarding specifically patients with COPD, a recent trial compared SBT strategies. Matic et al included 63 patients who had already a SBT failure and found a lower ICU length of stay using PS compared to T-Piece. Nevertheless, study design limitations and the non-standardization of non-invasive ventilation use limit the interpretation of these results.

Therefore, the best strategy to proceed MV liberation for patients with COPD is still unclear, warranting the performance of the present randomized controlled trial.

**3. Objectives:**

**3.1 Primary:**

To compare SBTs using PS with T-piece regarding MV duration, in patients with COPD mechanically ventilated for at least 48 hours.

**3.1 Secondaries:**

To compare, between the aformentioned SBTs, the following outcomes:

- ICU length of stay;
- Time to liberation from MV (weaning duration);
- Extubation failure rates;
- Mortality;
- Tracheostomy incidence.

**4. Equipoise:**

COPD is a commonly encountered comorbidity in patients with acute respiratory failure in ICUs worldwide, being associated with prolonged mechanical ventilation, increased length of stay and extubation failures.

Once extubation failure rates could be over 40% in this population, criterious assessment of which patients are suitable to MV liberation is warranted. This can be done by different ways, being SBTs with T-Piece and PS the most common approaches.

If SBTs through T-Piece seems to be more specific to detect extubation failures, this also can precipitate muscle fatigue and air trapping, specially in patients with dynamic airflow obstruction. In contrast, SBTs with PS intend to attenuate work of breathing and airway resistance, even if this does not reproduce actual post-extubation scenario.

Once this question is still unclear and the condition to be studied is highly prevalent, the performance of this trial is justified.

**5. Methods:**

**5.1 Study locations:**

- Hospital Nossa Senhora da Conceição: Intensive Care Unit

- Hospital de Clínicas de Porto Alegre: Intensive Care Unit

- Hospital Montenegro: Intensive Care Unit

**5.2 Patients:**

**5.2.1 Inclusion criteria:**

Consecutive patients with COPD will be included if older than 18 years, admitted do the participant ICUs and mechanically ventilated for at least 48 hours.

Diagnosis of COPD will be made by clinical assessment, review of medical recordsand biochemical and radiologial data.

**5.2.2 Exclusion criteria:**

- Tracheostomy installation before the start of MV liberation procedure;

- Refuse to deliver informed consent;

- Individuals allocated in another clinical trial.

**5.2.3 Design:**

Randomized controlled trial.

This study was previously registerd at ClinicalTrials.gov under registration number NCT01464567.

**5.2.4 Data assessment and randomization:**

Patient inclusion will be made only after individuals complete 48 hours from MV initiation, and after informed consent obtainance. Data will be collected in prespecified charts, omitting patients’ identification.

As soon as the individual is considered able to perform a SBT, the randomization procedure is performed, defining study group: SBT through T-Piece or SBT through PS. Randomization will be in blocks of 10, according to the SAPS III (simplified acute physiology score, version 3) score. Randomization will be done using sealed numbered opaque envelopes.

Patients allocated to the PS group will have inspiratory pressure level reduced to 10cmH2O to attenuate airway resistance imposed by the mechanical apparatus. Patients allocated to the T-piece group will have their endotracheal tubes disconnected from the ventilator and attached to a T-connector, which permits connection to an oxygen-enriched supply while breathing at room air pressure.

Patients will be continuously monitored according to heart and respiratory rate, arterial pressure and pulse oximetry. SBTs will have 30-minutes duration in both groups.

Extubation will be performed according to predefined criteria, as previously published: respiratory rate > 35 breaths/min, oxygen saturation < 88%, heart rate > 140/min, systolic blood pressure > 200 mmHg or < 80 mmHg (or any variation exceeding 30% difference from the baseline), exaggerated use of accessory respiratory muscles, consciousness disturbance, diaphoresis or new arrhythmia. Patients who failed SBT were returned to their previous MV settings for at least 24 hours. The same SBT randomized modality was maintained for further SBTs.

Following extubation, all patients will be preemptively connected to non-invasive mechanical ventilation.

**5.2.5 Statistical analysis:**

Data will be stored and analysed using the Software SPSS Statistics 18.0. Continuous variables will be presented as means and standard deviation or medians and interquartile intervals. Student’s t test or Wilcoxon-Mann-Whitney test will be used for comparisons. Categorical variables will be reported as percentages and compared by chi-square test or Fisher exact test, when appropriate.

Statistical significance level will be considered a two-tailed p-value lower than 0,05.

**5.2.6 Sample size calculation:**

We used the mean MV duration for individuals with COPD admitted to the ICU of Hospital Nossa Senhora da Conceição of 5.8 (± 2.44) days to estimate sample size. Therefore, 95 patients in each group must be included to detect a 1-day reduction, considering a study power of 80%.

**6. Ethical aspects:**

This study will be initiated only after Ethics commitee of Hospital Nossa Senhora da Conceição appreciation and approval.

Patients will be included only after informed consent is obtained. The informed consent form will be presented in two identical copies, being one of them left with the patient or the next of kin.

The principal investigator assures confidentiality concerning personal data obtained. All the informations will be exclusively analysed in an aggregated way, preserving the identity of all individuals. Data will be used only for this study, being stored for five years.

All the ethical aspects are in accordance with resolution CNS (national health council) 196/96. The investigators are committed to be beneficent, compromisign themselves to maximize benefits and minimize risks. It is assured that the proposed procedures will respect confidentiality and privacy.

It is noteworthy that the procedures to be permormed in this study are already commonly implemented in clinical practice.

**7. Publication:**

Study results will be submitted to peer-review with the intention to scientific publication.

**8. Budget:**

There is no additional cost from laboratory exams and/or medications. No medication will be prescribed for study procedures. The only intervention will be SBTs performance as above described. The only cost will be office materials.

There is no specific funding from research agencies or government or privante entities.
